# AGO CLIP-based imputation of potent siRNA sequences targeting SARS-CoV-2 with antifibrotic miRNA-like activity

**DOI:** 10.1038/s41598-021-98708-z

**Published:** 2021-09-27

**Authors:** Seung Hyun Ahn, Dowoon Gu, Yongjun Koh, Hye-Sook Lee, Sung Wook Chi

**Affiliations:** 1 Department of Life Sciences, Korea University, Seoul, Korea; 2 Division of Biotechnology, College of Life Sciences and Biotechnology, Korea University, Seoul, Korea

**Keywords:** RNAi therapy, Sequence annotation

## Abstract

Coronavirus disease 2019 (COVID-19), caused by severe acute respiratory syndrome coronavirus 2 (SARS-CoV-2), is associated with fatal pulmonary fibrosis. Small interfering RNAs (siRNAs) can be developed to induce RNA interference against SARS-CoV-2, and their susceptible target sites can be inferred by Argonaute crosslinking immunoprecipitation sequencing (AGO CLIP). Here, by reanalysing AGO CLIP data in RNA viruses, we delineated putative AGO binding in the conserved non-structural protein 12 (nsp12) region encoding RNA-dependent RNA polymerase (RdRP) in SARS-CoV-2. We utilised the inferred AGO binding to optimise the local RNA folding parameter to calculate target accessibility and predict all potent siRNA target sites in the SARS-CoV-2 genome, avoiding sequence variants. siRNAs loaded onto AGO also repressed seed (positions 2–8)-matched transcripts by acting as microRNAs (miRNAs). To utilise this, we further screened 13 potential siRNAs whose seed sequences were matched to known antifibrotic miRNAs and confirmed their miRNA-like activity. A miR-27-mimicking siRNA designed to target the nsp12 region (27/RdRP) was validated to silence a synthesised nsp12 RNA mimic in lung cell lines and function as an antifibrotic miR-27 in regulating target transcriptomes related to TGF-β signalling. siRNA sequences with an antifibrotic miRNA-like activity that could synergistically treat COVID-19 are available online (http://clip.korea.ac.kr/covid19).

## Introduction

Severe acute respiratory syndrome coronavirus 2 (SARS-CoV-2) is responsible for the current coronavirus disease 2019 (COVID-19) pandemic, where its rapid spread among humans continues to threaten global health^1^. The major risk of COVID-19 is fatal lung injury with acute respiratory distress syndrome which can progress to idiopathic pulmonary fibrosis, resulting in the loss of respiratory function^2^. Because there is no effective cure for COVID-19, antifibrotic drugs have been suggested to attenuate the profibrotic pathways in SARS-CoV-2 infection. Nevertheless, effective treatment of COVID-19 requires both inactivation of SARS-CoV-2 infection and prevention of progression to lung fibrosis.

SARS-CoV-2 consists of a positive-sense single-stranded genomic RNA (gRNA) of ~ 29,800 bases^1^. Its entry into cells is mediated by the interaction between its surface spike (S) protein and the angiotensin-converting enzyme 2 of the host^3^. The infecting gRNA is then translated into polypeptides from two open reading frames (ORF1a and ORF1b) and further cleaved into 16 non-structural proteins (nsps) via proteolysis domains^4^. To achieve viral replication, the gRNA in the positive sense should be copied by RNA-dependent RNA polymerase (RdRP) into negative-sense gRNA, which serves as a template for replication. The RdRP of SARS-CoV-2 is encoded in nsp12 and is essential for SARS-CoV-2 life cycle^5^. Thus, RdRP has often been therapeutically targeted for the development of antiviral drugs (e.g. Remdesivir^6^).

Intrinsically, host cells contain several antiviral defence mechanisms and one of such is RNA interference (RNAi)^7,8^. Small interfering RNA (siRNA) can induce RNA interference (RNAi) for a specific target gene by loading onto Argonaute (AGO), triggering cleavage of the target mRNA via perfect sequence complementarity^9^ and many organisms, including mammals, use virus-derived siRNAs to induce RNAi against viruses^7,8^. Since SARS-CoV-2 harbours single-stranded RNA as a genome, it is designated to be susceptible to RNAi as shown in other RNA viruses (e.g. TKM-Ebola^10^, the siRNA drug against Ebola virus). Therefore, siRNAs targeting SARS-CoV-2 are advantageous for development as naturally effective and specific therapeutics^11^.

Upon viral infection, endogenous small non-coding RNAs, microRNAs (miRNAs), can also trigger antiviral RNAi^12^: miRNAs loaded on AGO recognise target transcripts primarily by base-pairing with the seed region (positions 2–8) and suppress hundreds of target transcripts by reducing mRNA stability and/or translation^13^. Relevant to antiviral activity, miRNAs exert various pathophysiological functions depending on the roles of the target mRNAs in the biological pathways. For instance, miRNAs, such as miR-27^14,15^, miR-29^16^, miR-486, and miR-455^17^, have been reported to attenuate lung fibrosis by targeting multiple genes in the TGF-β signalling pathways, of which activation potentiates pulmonary fibrosis upon viral infection^2^. Therefore, the antifibrotic activity of these miRNAs may be beneficial for treating COVID-19.

siRNAs loaded on AGO can also function similarly to miRNAs, suppressing hundreds of off-target transcripts wherever the seed region can make a match^18^. Thus, miRNA-like off-targets must be evaluated and controlled^9^, where chemical modifications, including abasic pivot substitution^19,20^, have successfully prevented the off-targeting^21^. However, a miRNA-mimicking activity could be advantageously arranged in siRNAs by containing the same seed sequences of miRNAs with specific functions^22^, as shown in the development of synergistic anticancer siRNA drugs^23^.

Since both siRNA and miRNA share the same RNAi effector, AGO, it is important to map AGO binding on the global transcriptome to understand their positional occupancy and pathophysiological function. For this, sequencing of AGO-associated RNAs isolated via crosslinking immunoprecipitation (AGO CLIP)^24^ has been developed with bioinformatics analyses^25^, proven to be sensitive enough to discover miRNA responsive elements including canonical and non-canonical miRNA target sites^26,27^ as well as those recognised by miRNA oxidation^28^. Upon viral infection, AGO CLIP has helped researchers understand miRNA targets and their pathological roles in 15 different RNA viruses^29^. Moreover, AGO-bound miRNA target sites analysed using CLIP could define accessible target regions for RNAi^23,30^, enabling us to overcome the variable susceptibility of siRNAs, which can be estimated by calculating the exposure probability of RNA folding because structural accessibility of the target mRNAs can affect siRNAs^31^. Therefore, based on AGO CLIP results, it could be feasible to design potent siRNA against SARS-CoV-2 as a primary target (preferentially targeting critical regions such as RdRP), which could also act with a miRNA-like mechanism to inhibit fibrosis (inhibiting the same targets as antifibrotic miRNAs as secondary targets). Thus, these siRNAs should inhibit both SARS-CoV-2 replication and associated pulmonary fibrosis.

In line with this, we designed potential siRNA sequences harbouring seed matches of known antifibrotic miRNAs (miR-27a, miR-193-5p, miR-486, miR-151, and miR-455)^17^. We inferred AGO-accessible regions in the conserved RdRP (nsp12) region in SARS-CoV-2 through a meta-analysis of published AGO CLIP data in other RNA viruses^29^ and further utilised them to precisely calculate exposure probability in the local folding of SARS-CoV-2 gRNA. By selecting antifibrotic miRNA seed sequences in the accessible siRNA target sites and avoiding the sequence variation of SARS-CoV-2 in the human spread, we designed 13 potent antifibrotic siRNAs. Specifically, miR-27 seed containing siRNA against RdRP (27/RdRP) was experimentally validated and its antifibrotic activity was confirmed using RNA sequencing (RNA-Seq) analyses. Overall, we set out to use AGO CLIP analysis to design potential siRNA drugs that silence SARS-CoV-2 and exhibit antifibrotic miRNA activity for the treatment of COVID-19.

## Material and methods

### Bioinformatics and statistical analyses

We used Python scripts (Biopython; https://biopython.org/) and Integrative Genomics Viewer (IGV; https://software.broadinstitute.org/software/igv/) for bioinformatics analysis. RNA-Seq analysis was performed using Cufflinks and Cuffdiff (http://cole-trapnell-lab.github.io/cufflinks/).

A standard laboratory practice randomisation procedure was used for all experimental groups. The investigators were not blinded to the allocations assigned during the experiments and the outcome assessment. All statistical tests, including the Wilcoxon rank-sum test (two-sided), Kolmogorov–Smirnov test (two-sided), *t*-test (unpaired two-tailed), and chi-square test were conducted using R (https://www.r-project.org/), SciPy (http://www.scipy.org/), or Excel. The values are presented as mean ± SD. Statistical significance was set at *P*-value = 0.05, relative to control, equal variance, unless stated otherwise; repeated with biologically independent samples (n ≥ 3).

### Sequence analysis of SARS-CoV-2

Different sequencing results of SARS-CoV-2 gRNAs from the infected human host, currently deposited in the GenBank (n = 5475; 7th June, 2020), were downloaded (https://www.ncbi.nlm.nih.gov/sars-cov-2/), aligned using the multiple sequence alignment program, MAFFT (https://mafft.cbrc.jp/alignment/software/), and used to calculate the conservation rate (%) at each position via a sliding 6 nucleotide window, in which the size reflects the minimum length of the miRNA seed. The conservation rates were visualised with the positions in SARS-CoV-2 gRNA (NC_045512; reference sequence in GenBank) using IGV.

### Meta-analysis of AGO CLIP data for SARS-CoV-2

RNA sequences encoding the RdRP of RNA viruses were retrieved from the GenBank for SARS-CoV-2 (NC_045512, positions 13442–16236; nsp12), HCV (NC_009823, positions 7667–9439; NS5B), HAV (M59808, positions 5929–7395; 3D), CVB (NC_038307, positions 5912–7297; 3Dpol), SINV (NC_001547, positions 5751–7598; nsP4), CHIKV (NC_004162, positions 5648–7498; nsP4), and VEEV (NC_001449, positions 5703–7520; nsP4). All sequences were analysed using the Clustal Omega program (https://www.ebi.ac.uk/Tools/msa/clustalo/) to achieve multiple sequence alignments. These alignments were further applied to generate neighbouring phylogenetic trees using the PHYLIP package (DNAdist and Protdist; https://evolution.genetics.washington.edu/phylip.html). Based on the phylogenetic analysis results, RNA viruses related to the nsp12 sequence of SARS-CoV-2 were selected.

For the meta-analysis of the AGO CLIP data, pairwise sequence alignment of the nsp12 region in SARS-CoV-2 was initially performed for each RdRP region, as described above for different viruses (i.e. where AGO CLIP experiments have been performed in infected host cells)^29^. Of note, since Ago2 antibodies used in the experiments^29^ were proven to be anti-pan Ago antibodies^24^, we referred them as data from AGO. After directly retrieving AGO CLIP data^29^, originally derived from the GEO database for HCV (n = 7; GSM2041566, GSM2041567, GSM2041568, GSM2041569, GSM2041570, GSM2041571, GSM2041572), HAV (n = 7; GSM2041645, GSM2041646, GSM2041647, GSM2041648, GSM2041649, GSM2041650, GSM2041651), CVB (n = 4; GSM2041622, GSM2041623, GSM2041624, GSM2041625), SINV (n = 6; GSM2041626, GSM2041627, GSM2041628, GSM2041629, GSM2041630, GSM2041631), CHIKV (n = 4; GSM2041632, GSM2041633, GSM2041636, GSM2041637), and VEEV (n = 2; GSM2041640, GSM2041641), coordination of the compiled read-counts mapped onto the viral genome^29^, were lifted to the nsp12 region of SARS-CoV-2 using the pairwise sequence alignment results; the counts were then used to generate bedGraph files and further analysed using IGV.

### Calculation of the exposure probability in SARS-CoV-2

The exposure probability (P_exp_) of the siRNA target sites was determined using the RNAplfold program (https://www.tbi.univie.ac.at/RNA/RNAplfold.1.html) as previously described^31^. The partition function for all local structures within the 80 nucleotide-long window (*W* = 80, as previously defined as optimal)^31^ was calculated to derive the accessibility (log_10_(P_exp_)) of a target site in a predefined length (*u*) within the specified maximal distance (*L*) between two base-pairing positions. To select the optimal folding parameters, the difference in exposure probability (ΔP_exp_) between two sets of AGO-bound and either AGO-unbound or total regions (defined in nsp12 of SARS-CoV-2 by the meta-analysis of AGO CLIP data) was examined to consider the length of the seed site (*u* = 6, 7, and 8) with different constraints (*L* = 20, 40, and 60), compared with the previously defined length (*u* = 16) for siRNA target accessibility^31^. *P*-values were calculated using the Wilcoxon rank-sum test (two-sided) in R. To design siRNAs with seed sequences (6mers or more in positions 2–8) of antifibrotic miRNAs (miR-27, miR-193a-5p, miR-486, miR-151, and miR-455)^17^, target sites were selected in the siRNA-accessible regions (P_exp_ > 0.1) of SARS-CoV-2 gRNA (NC_045512) based on calculations using the optimised parameters (*u* = 8 and *L* = 20).

### RNA synthesis

siRNA and miRNA were synthesised (Supplementary Table S1) using custom RNA synthesis services provided by Bioneer (Korea). The quality of the synthesised RNAs and their modifications were monitored, reported, and confirmed by the manufacturer. To serve as a negative control, non-targeting miRNA (NT) derived from the cel-miR-67 sequence (*Caenorhabditis elegans*-specific miRNA) was synthesised as an siRNA with two thymidine deoxynucleotide (dT) overhang. To avoid miRNA-like repression, the siRNA was further modified to contain abasic pivot (abasic deoxynucleotide, dSpacer (Ø), at position 6) in both strands as previously reported^19,20^. Duplexes of the miRNA, miR-27a, and miR-193a-5p, were synthesised according to the human miRNA annotation in miRBase (http://www.mirbase.org/), with consideration of the two-nucleotide overhang. For RNA-Seq analysis, the miR-27a sequence was synthesised according to the annotation in miRbase; however, its passenger strands contain 2ʹ-O methylation at positions 1 and 2 (2ʹOMe) in the form of siRNA, preventing seed-mediated repression from the passenger strand. siRNAs designed to contain the miR-27a seed sequence were synthesised as indicated in Fig. 3d; these siRNAs also contain 2ʹOMe in the passenger strands. Details of the siRNA and miRNA sequences are provided in Supplementary Table S1.

### Cell culture, transfection, and treatment

The human epithelial lung carcinoma cell line, A549 (Korean Cell Line Bank), was grown in RPMI-1640 (Hyclone). The human fibroblast lung cell line MRC-5 (Korean Cell Line Bank) was grown in MEM (Hyclone). HeLa cells (Korean Cell Line Bank) were grown in Dulbecco’s modified Eagle’s medium (DMEM; Hyclone). All media were supplemented with 10% fetal bovine serum (FBS; Gibco), 100 U·mL^−1^ penicillin, and 100 μg·mL^−1^ streptomycin (Welgene) and incubated at 37 °C with 5% CO_2_.

Unless otherwise indicated, transfection of siRNA or miRNA (50 nM) was performed using Lipofectamine RNAiMAX (Invitrogen) according to the general protocol provided by the manufacturer. Lipofectamine 3000 (Invitrogen) was used to co-transfect the RNA with plasmid vectors according to the manufacturer’s protocol. To seed cells prior to transfection, the number of cells was quantified using the Countess II Automated Cell Counter (Invitrogen). The cells were harvested 24 h after transfection unless otherwise indicated. To induce differentiation of the fibroblast lung cell line, MRC-5, 200,000 cells per well in 6 well plates were transfected with 50 nM RNA using Lipofectamine RNAiMAX (Invitrogen) and then 2.5 ng/mL of human recombinant transforming growth factor-β1 (TGF-β, Sigma-Aldrich) was added after 24 h. Cell morphology was examined using inverted microscopy (Leica DMi8).

### Construction of luciferase reporter constructs

To measure the efficiency of RNAi-mediated repression, the psiCheck-2 vector (Promega) was used to construct luciferase reporters, which had an on-target site inserted into the 3′ untranslated region (3′UTR) of Renilla luciferase. Accordingly, synthetic duplex DNA oligos (Bionics, Korea) containing different on-target sites (bold) were cloned into the psiCheck-2 plasmid via the XhoI and NotI sites as indicated: 151/nsp3, forward: 5′-TCGAGATGAAGTTGCGAGAGACTTGTCACTACAGTTTAAAAGACCAATAA**ATCCTACTGACCAGTCTTC**TTACATCGTTGATAGTGTTACAGTGAAGAATGGTTCCATCCATCTGC-3′, reverse: 5′-GGCCGCAGATGGATGGAACCATTCTTCACTGTAACACTATCAACGATGTAA**GAAGACTGGTCAGTAGGAT**TTATTGGTCTTTTAAACTGTAGTGACAAGTCTCTCGCAACTTCATC-3′; 193/nsp3, forward: 5′-TCGAGGCTGGTAGTACATTTATTAGTGATGAAGTTGCGAGAGACTTGTCA**CTACAGTTTAAAAGACCAA**TAAATCCTACTGACCAGTCTTCTTACATCGTTGATAGTGTTACAGGC-3′, reverse: 5′-GGCCGCCTGTAACACTATCAACGATGTAAGAAGACTGGTCAGTAGGATTTA**TTGGTCTTTTAAACTGTAG**TGACAAGTCTCTCGCAACTTCATCACTAATAAATGTACTACCAGCC-3′; 193/nsp5, forward: 5′-TCGAGAACTCTTAATGACTTTAACCTTGTGGCTATGAAGTACAATTATGA**ACCTCTAACACAAGACCAT**GTTGACATACTAGGACCTCTTTCTGCTCAAACTGGAATTGCCGTTGC-3′, reverse: 5′-GGCCGCAACGGCAATTCCAGTTTGAGCAGAAAGAGGTCCTAGTATGTCAAC**ATGGTCTTGTGTTAGAGGT**TCATAATTGTACTTCATAGCCACAAGGTTAAAGTCATTAAGAGTTC-3′; 151-seed, forward: 5′-TCGAG**CAGTCT**AA**CAGTCT**ATA**CAGTCT**AAT**CAGTCT**AAA**CAGTCT**ATGC-3′, reverse: 5′-GGCCGCAT**AGACTG**TTT**AGACTG**ATT**AGACTG**TAT**AGACTG**TT**AGACTG**C-3′; 486/RdRP, forward: 5′-TCGAG**ACTGATGTCGTATACAGGA**GC-3′, reverse: 5′-GGCCGC**TCCTGTATACGACATCAGT**C-3′; 27/RdRP, forward: 5′-TCGAG**AACAGTACAATTCTGTGAA**GC-3′, reverse: 5′-GGCCGC**TTCACAGAATTGTACTGTT**C-3′.

### Luciferase reporter assays

Luciferase reporter assays were performed as previously described^19^. Briefly, psiCheck-2 plasmids (Promega) were co-transfected with different amounts of duplex RNA (up to 100 nM) using Lipofectamine 3000 (Invitrogen). Twenty-four hours after transfection, relative activity (Renilla luciferase activity normalised to firefly luciferase) was measured using the Dual-Luciferase Reporter Assay System (Promega) and the GloMax-Multi Detection System (Promega), with replicates (n = 6), according to the manufacturer’s protocol. The half-maximal inhibitory concentration (IC_50_) was calculated via nonlinear least-squares fitting for the sigmoid function using SciPy (scipy.optimize.curve_fit()). When the least-squares failed to fit the function, the approximate IC_50_ was estimated from the regression line.

### RNA-Seq analysis

RNA-Seq libraries were constructed from a large RNA using the strand-displacement stop/ligation method. Briefly, 1.5 μg of large RNA (extracted using RNeasy Mini Kit; Qiagen), polyadenylated mRNA was extracted with 10 μL of Dynabead Oligo(dT)_25_ (Invitrogen), according to the manufacturer’s protocol. Thereafter, 10 ng of the purified mRNA was subjected to RNA-Seq library preparation using the CORALL Total RNA-Seq Library Prep Kit (Lexogen), according to the manufacturer’s instructions. After the cDNA was constructed, the libraries were amplified using PCR with the lowest optimal cycle, which was determined by performing qPCR (Rotor-Gene Q; Qiagen) with the addition of SYBR Geen I (1:10,000; Invitrogen) to the Q5 High-fidelity 2 × master mix (New England Biolabs). The quality of the amplified library was verified using Fragment Analyzer (Advanced Analytical) and the size distribution and quantity were estimated. After cross-checking the concentration of libraries using the Qubit DNA HS assay kit (Thermo Fisher Scientific), the prepared multiplexed libraries were pooled and sequenced as 75 single-end reads (SE75) using the MiniSeq system (Illumina). All sequence data were deposited in the SRA database (SRP270828).

The de-multiplexed sequencing reads were aligned with the human genome (hg19) using STAR (star—outSAMtype BAM SortedByCoordinate—outSAMattrIHstart 0—outFilterType BySJout—outFilterMultimapNmax 20—outFilterIntronMotifs RemoveNoncanonicalUnannotated—outMultimapperOrder Random—alignSJoverhangMin 8—alignSJDBoverhangMin 1) with RefSeq gene annotations. The first 12 nucleotides from the starter side of the reads were trimmed before mapping as instructed by the manufacturer.

Transcript abundance was quantified as reads per kilobase of transcript per million mapped reads (RPKM) using Cufflinks (cufflinks -b -G—compatible-hits-norm—library-type fr-secondstrand), and the statistical significance of the differential expression (*P*-value) was derived using Cuffdiff (Cuffdiff—FDR = 0.1 -b—compatible-hits-norm—library-type fr-secondstrand), with a supply of mapping results in groups under the same experimental conditions. Only values with a valid status were selected and further analysed to identify differentially expressed genes (DEGs) with statistical significance (*P*-value).

### CDF and volcano plot analyses

To examine the propensity of miRNA-like target repression depending on the transfection of a given duplex RNA, the cumulative distribution function (CDF) according to fold change (log_2_ ratio) was analysed using RPKM values derived from Cufflinks. Putative miRNA targets were selected if they contained cognate seed sites in the 3′UTR (annotated by RefSeq, downloaded from the UCSC genome browser), and the 7mer-A1 seed site (match to positions 2–8 with A at position 1) was generally searched unless otherwise indicated. “No site” indicated transcripts that had no cognate 6mer sites from the seed (positions 2–8) in their mRNA sequences. “Cont site” denoted that the subset of “No site” transcripts with a control site in the 3′UTR (i.e. where the nucleotide should align with pivot (position 6) was substituted by the same nucleotide to achieve impairment^26,27^). Total mRNAs were selected for the expression of mRNAs (Cufflinks; RPKM ≥ 0.1). The KS test was performed using SciPy (scypy.stats.ks_2samp()) relative to total mRNAs or control sites. The volcano plot was analysed by calculating the fold change and significance (− log_10_ (*p*-value)) derived from Cuffdiff; the cutoffs were used to select the DEGs.

### Gene ontology analysis

Gene ontology (GO) analysis was performed using DAVID (http://david.abcc.ncifcrf.gov/), with a supply of downregulated DEGs (selected by volcano plot analysis) under the background of expressed transcripts in a matched control set (selected as RPKM > 0 and log_2_ (volume) > 2, where volume is the average RPKM across different conditions), and default parameters were employed unless otherwise indicated. GO analysis results (EASE score < 0.15, or 0.2) were visualised using REVIGO (http://revigo.irb.hr/) in the network of biological process terms. An interconnected graph, which had highly similar GO terms linked to represent the degree of similarity based on width, was also analysed.

### Overlap analysis of 27/RdRP and miR-27a downregulated targets

In RNA-Seq data, downregulated target transcripts (*P* < 0.05 or log_2_ fold change < -0.5) depending on 27/RdRP or miR-27a expression (6mer (positions 2–7) seed site in the 3′UTR) were selected from the total expressed transcripts (RPKM > 0 and log_2_ (volume) > 2). To determine the statistical significance of the overlap between the 27/RdRP- and miR-27a-dependent target transcripts, the average number of random overlaps (1000 iterations of random shuffling in DEGs using Python, random.shuffle()) was calculated to determine the *P*-value using the chi-square test (scipy.stats.chisquare). GO analysis of the overlapping target transcripts was performed using DAVID, with the background of expressed transcripts in both 27/RdRP and miR-27a (selected as RPKM > 0 and log_2_ (volume) > 2). GO analysis results were visualized by REVIGO (EASE score < 0.2) or clustered with low classification stringency (presented in Supplementary Table S2).

### Prediction of the SARS-CoV-2 nsp12 secondary structure

The SARS-CoV-2 nsp12 gRNA sequence was downloaded from the GenBank (NC_045512:13442–16236), and the secondary structure was predicted using the mfold program (version 3.6)^32^ with default parameters. The minimum free energy of 27/RdRP and 486/RdRP seed regions (positions 2–8) that hybridise with target sites in the nsp12, was calculated using RNAduplex (version 2.4.15)^33^.

### Quantitative RT–PCR analysis

Total RNA was isolated using the RNeasy Mini Kit (Qiagen) via on-column DNA digestion with the RNase-Free DNase Set (Qiagen). Reverse transcription was performed using SuperScript III Reverse Transcriptase (Invitrogen) and oligo(dT) primers, qPCR was performed using the QuantiNova SYBR Green PCR Kit (Qiagen) and custom primers for COL1A1 mRNA (forward primer: 5′-GCCAAGACGAAGACATCCCA-3′, reverse: 5′-CGTCATCGCACAACACCTTG-3′) on Rotor-Gene Q (Qiagen). All reactions were run in triplicate with the standard two-step cycling protocol. Relative quantification was performed using the ΔCT method, with GAPDH (forward: 5′-TGCACCACCAACTGCTTAGC-3′, reverse: 5′-GGCATGGACTGTGGTCATGAG-3′).

### Quantification of SARS-CoV-2 target RNAs silenced by siRNA

To prepare the nsp12 transcript, we initially synthesised the DNA of the nsp12 transcript (NC_045512, positions 13442–16236; ~ 2.8 kb), whose 5′ end was fused with the T7 promoter (5′-GGATCCTAATACGACTCACTATAGG-3′) using a custom synthesis service provided by Bionics (Korea). The product was further amplified using PCR (forward primer, 5′-GGATCCTAATACGACTCACTAT-3′; reverse primer, 5′-CTGTAAGACTGTATGCGGTG-3′) using Q5 High-Fidelity 2 × Master Mix (New England Biolabs). To serve as a control transcript that harbours no target site, we produced a T7 promoter-containing DNA of codon-optimised nsp12, amplified from a pLVX-EF1alpha-SARS-CoV-2-nsp12-2 × Strep-IRES-Puro plasmid (Addgene plasmid # 141378, positions 3550–6453) using PCR (forward primer, 5′-GGATCCTAATACGACTCACTATAGGATGTCAGCAGACGCACAAAG-3′; reverse primer, 5′-CTGCAGGACGGTGTGAGGC-3′). After purification using the QIAquick Gel Extraction kit (Qiagen), the DNA was used as a template for in vitro transcription, which was performed using the T7 RiboMAX Express RNAi System (Promega) according to protocols provided by the manufacturer’s instructions. After the DNA templates were digested via treatment with RQ1 RNase-Free DNase (Promega) at 37 °C for 30 min, the synthesised RNAs were purified using RNA Clean & Concentrator Kit (Zymo Research) and further polyadenylated using a Poly(A) Tailing Kit (Invitrogen), according to the manufacturer's instructions.

After purification with RNA Clean & Concentrator Kits (Zymo Research), the extracted RNA transcripts (250 ng) were co-transfected with 50 nM siRNA into A549 cells (0.2 × 10^6^ cells) using Lipofectamine 3000 (Invitrogen), according to the manufacturer’s protocol. After 24 h, total RNA was purified using QIAzol (Qiagen), followed by isopropanol precipitation. The amount of transfected RNA was measured using qPCR. Reverse transcription was performed using SuperScript III Reverse Transcriptase (Invitrogen) and oligo(dT) primers. qPCR was conducted using the QuantiNova SYBR Green PCR Kit (Qiagen) and custom primers (nsp12, forward primer: 5′-AGGTGAACGTGTACGCCAAG-3′, reverse primer: 5′-CTGGCGTGGTTTGTATGAAA-3′; codon-optimised nsp12, forward primer: 5′-GACTTCGTCGAAAACCCTGA-3′, reverse primer: 5′-CGTAAGCACACCAACAATGC-3′) using Rotor-Gene Q (Qiagen). All reactions were run in triplicate with the standard two-step cycling protocol. Relative quantification was calculated using the ΔCT method, with nsp12 normalised by the codon-optimised nsp12.

To compare the activity of two siRNAs (27/RdRP and 486/RdRP) that target nsp12 of SARS-CoV-12, the RNA fragment containing each on-target site (27/RdRP or 486/RdRP) with ± 43 nucleotides flanking regions was produced using in vitro transcription. Briefly, DNA fragments of the SARS-CoV-2 RdRP (NC_045512; positions 13961–14065, 27/RdRP; positions 13479–13583, 486/RdRP) under the T7 promoter (5′-GGATCCTAATACGACTCACTATAGG-3′) were synthesised (Bionics, Korea) and used as templates for the T7 RiboMAX Express RNAi System (Promega). After purification using QIAzol (Qiagen) and isopropanol precipitation, the synthesised RNA fragments (60 ng) were co-transfected with 50 nM siRNA into A549 cells (0.2 × 10^6^ cells) using Lipofectamine 3000 (Invitrogen) according to the manufacturer’s protocol. After 24 h, the cells were harvested for small RNA (< 200 nucleotides) isolation using the miRNeasy Mini kit (Qiagen). The purified small RNAs were then reverse transcribed using SuperScript III Reverse Transcriptase (Invitrogen) with specific primers for RNA fragments harbouring 27/RdRP (5′-CTAATGTCAGTACACCAACA-3′) or 486/RdRP (5′-TTAGCAAAACCAGCTACTTTATC-3′). The relative amount of RNA fragments containing 27/RdRP vs.486/RdRP was measured using qPCR. qPCR was performed using the QuantiFast SYBR Green PCR Kit (Qiagen) and custom primers (27/RdRP, forward primer: 5′-TACGCCAACTTAGGTGAACG-3′, reverse primer: 5′-CCAACAATACCAGCATTTCG-3′; 486/RdRP, forward primer: 5′-GTGTAAGTGCAGCCCGTCTT-3′, reverse primer: 5′-TGTCAAAAGCCCTGTATACGAC-3′) using Rotor-Gene Q (Qiagen). All qPCR reactions were performed in triplicate using the standard two-step cycling protocol. Relative quantification was performed using the ΔΔCT method. Of note, the 486/RdRP fragment was used as a control for the 27/RdRP fragment and vice versa.

### Construction of the siRNA webserver for SARS-CoV-2

The siRNA webserver for SARS-CoV-2 (http://clip.korea.ac.kr/covid19) was constructed using the Django framework (https://www.djangoproject.com/) in Python and MySQL. The web interface was implemented using HTML, CSS, and JavaScript components, where the JavaScript components utilised the JQuery library (http://jquery.com/).

## Results and discussion

### AGO CLIP-based imputation of AGO accessibility in SARS-CoV-2

**Figure 1:**
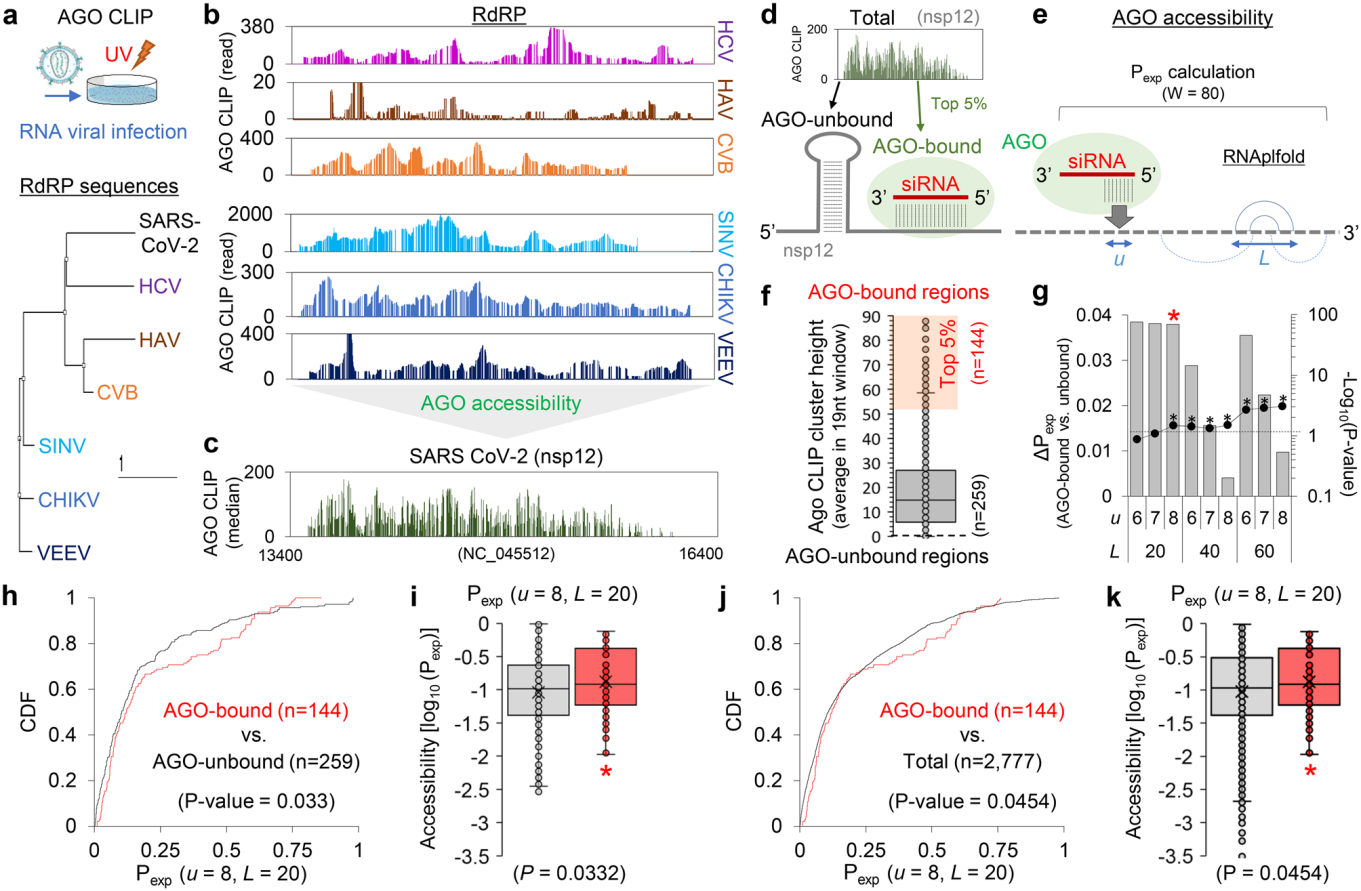
Meta-analyses of AGO CLIP data for SARS-CoV-2 and resultant determination of parameters for calculating AGO accessible regions. (**a**) AGO CLIP analyses derived from various RNA viral infections (upper panel), where published data^29^ from standard AGO CLIP were retrieved. Six RNA viruses were selected as related to SARS-CoV-2 based on phylogenic analyses of RdRP sequences (lower panel). Details in the Material and methods section, providing accession numbers in the GEO database. (**b**) AGO-bound regions in the RdRP of the six RNA viruses related to SARS-CoV-2, aligned on the counterpart region of the nsp12 in SARS-CoV-2; HCV, HAV, and CVB (**b**, upper panel) and SINV, CHIKV, and VEEV (**b**, lower panel) (**c**) Consensus AGO CLIP reads on the nsp12 of SARS-CoV-2 (NC_045512), delineated from the meta-analysis. Compiled AGO CLIP reads (median of read-counts at each position) are presented to estimate putative AGO binding in the nsp12 region of SARS-CoV-2, inferred from the six RNA viruses^29^. (**d,e**) Schematics for predicting AGO accessibility; AGO-bound (top 5%) and AGO-unbound (no CLIP reads) regions presumably caused by RNA secondary structure (**d**); calculation of AGO accessibility (P_exp_; exposure probability) using the previously defined window size (80 nucleotides; W = 80) under the parameters^31^, length of a region for accessibility (*u*) and range of nucleotides allowing local base pairing (*L*). (**f**) Ranking of the inferred AGO-accessible sites in nsp12. A set of high confident AGO-bound regions (high AGO accessibility) was defined by selecting the top 5% of compiled AGO CLIP clusters depending on their heights (average read-count in the 19 nucleotides window; n = 144, orange shade). AGO-unbound regions were selected as the sites with no AGO CLIP reads across the selected RNA viruses (HCV, HAV, CVB, SINK, CHIKV, and VEEV) or where AGO CLIP reads were observed in only one RNA virus species (biological complexity = 1; n = 259). (**g**) The partition function for all local structures within the window (80 nucleotides) was calculated to derive the exposure probability (P_exp_) with RNAplfold^31^. The difference in P_exp_ between the AGO-bound regions and AGO-unbound regions in nsp12 was calculated (ΔP_exp_, bar graph); significance, − log_10_ (*P*-value), line graph; dotted-line, *P* = 0.05; *P*-value, Wilcoxon rank-sum test (two-sided). (**h**) Cumulative distribution function (CDF) analyses of P_exp_ under the optimised parameter (*u* = 8, *L* = 20) for the AGO-bound vs. AGO-unbound regions; *P*-values from Kolmogorov–Smirnov test (two-sided). (**i**) Accessibility of the target sites (log_10_(P_exp_)) was also calculated for the AGO-bound (red box plot) vs. AGO-unbound regions (grey box plot); *P < 0.05, Wilcoxon rank-sum test (two-sided). (**j,k**) Same CDF analyses (**h**) performed in (**f**) and box plot analyses (**i**) performed in (**g**) except that the AGO-bound regions were compared with the total regions in nsp12.

To treat COVID-19, we designed potent siRNA sequences against SARS-CoV-2. To ensure the efficacy of RNA silencing, we decided to consider AGO target site accessibility in SARS-CoV-2 gRNA, which could be inferred from the AGO CLIP results of another RNA viral infection^29^ due to sequence similarity (Fig. 1a). In particular, focusing on the most conserved region of RNA viruses that encode RNA-dependent RNA polymerase (RdRP)^34^, we selected AGO CLIP results from the relevant RNA viruses categorised as having sequence similarity with SARS-CoV-2 by estimating the phylogenetic distance (Fig. 1a, lower panel). Then, we compiled the selected AGO CLIP reads on the SARS-CoV-2 RdRP gene (nsp12; Fig. 1b,c), where hepatovirus (HAV) and enterovirus B (CVB), which belong to the same *Pisoniviricetes* class as SARS-CoV-2, and hepatitis C virus (HCV) were chosen for primary alignment (Fig. 1b, upper panel), in addition to distantly related viruses of the family *Togaviridae* (Sindbis virus, SINV; Chikungunya virus, CHIKV; Venezuelan equine encephalitis virus, VEEV; Fig. 1b, lower panel). Since similar patterns of AGO-bound regions were observed throughout the related RNA viruses, we integrated their AGO CLIP reads into consensus AGO binding, which is potentially preserved in the conserved sequences, particularly coding for the critical RdRP gene, and finally enabled the imputation of AGO accessibility in the nsp12 region without performing biochemical CLIP experiments for SARS-CoV-2 (Fig. 1c).

Except for this conserved nsp12 region, AGO binding in SARS-CoV-2 could not be derived from the AGO CLIP data of other RNA viruses^29^. Thus, we should rather predict AGO accessibility based on local RNA folding, where the calculation of probability in a single-strand site could be optimised by using the deduced AGO binding in nsp12 (Fig. 1d,e). With this notion, we initially attempted to calculate the exposure probability (P_exp_) of local RNA folding by sliding 80 nucleotide-long windows (W = 80) as previously described (Fig. 1e)^31^. Because base pairing to seed (positions 2–8) initiates AGO target interaction^13^, we first specified the length of an exposed site within the seed region (*u* = 6, 7, or 8) and compared it to another by using a predefined length (*u* = 16) for siRNA from the previous study^31^. We also employed different ranges of maximum local base pairing (L = 20, 40, and 60) to calculate the target accessibility (log_10_ (P_exp_)). By comparing the predicted AGO-bound regions (top 5%) with AGO-unbound regions (no reproducible AGO CLIP read; Fig. 1f and Supplementary Fig. S1a) in nsp12, which were inferred by reanalysing AGO CLIP data from the related RNA viruses (Fig. 1a–e), we identified a set of optimal parameters, where the P_exp_ of the entire seed site (*u* = 8) within the range of ~ 20 nucleotide-long siRNAs (*L* = 20) outperformed other constraints (Fig. 1g, Supplementary Fig. S1b–e).

The results from the optimised parameter showed the greatest difference in average P_exp_ values between the AGO-bound and AGO-unbound regions (ΔP_exp_ = 0.038) in nsp12, among those that displayed statistical significance (− log_10_ (*P*-value) > 1.3; *P* < 0.05, Wilcoxon rank-sum test; Fig. 1g and Supplementary Fig. S1b). Under the optimised parameter (*u* = 8, *L* = 20), the same distinct difference was observed in the cumulative distribution function (CDF) of P_exp_ (Fig. 1h), as shown in the distribution of target accessibility (*P* = 0.033, Wilcoxon rank-sum test; Fig. 1i), where neither expanding the range of base pairing (*u* = 8, *L* = 40; Supplementary Fig. S1f.) nor considering the accessibility of almost the entire length of siRNA (*u* = 16, *L* = 20 or 40; Supplementary Fig. S1g,h) improved the performance. When AGO-bound regions (defined as the top 5%) were compared with the total, the same distinct difference was significantly observed (*P* = 0.045) by the optimised parameter (*u* = 8, *L* = 20; Fig. 1j,k) rather than others (Supplementary Fig. S2), supporting the increased accuracy of predicting AGO accessibility with this optimised parameter.

### Designing potent SARS-CoV-2 siRNAs with antifibrotic miRNA-like activity

**Figure 2:**
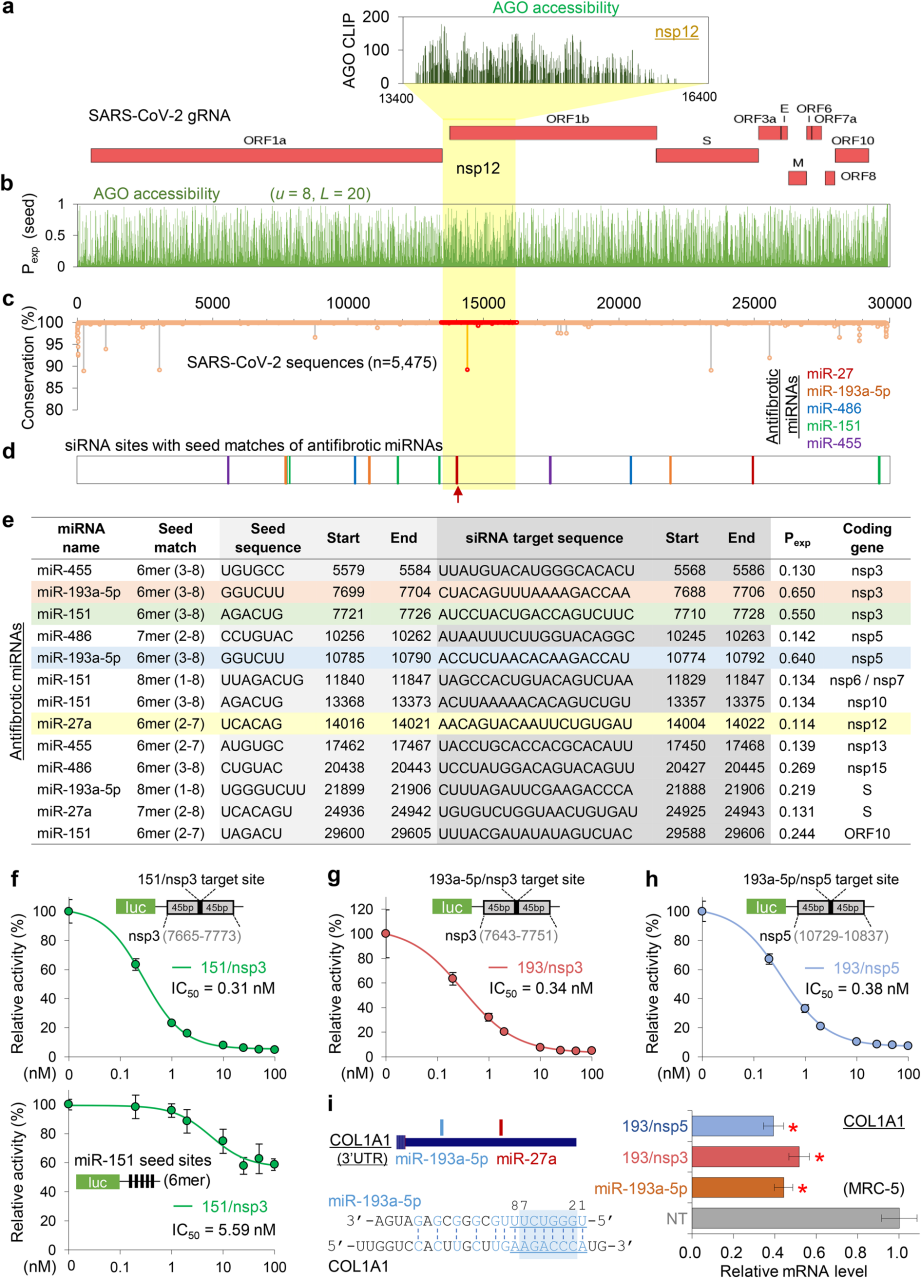
Putative AGO accessible regions in SARS-CoV-2 and the design of potent siRNAs containing the seed sequences of antifibrotic miRNAs. (**a**) Putative AGO binding in the nsp12 region of SARS-CoV-2 was presented as the density of AGO CLIP reads, inferred from the RdRP regions of the related RNA viruses (upper panel). The structure of SARS-CoV-2 gRNA was presented (lower panel). (**b**) Susceptible siRNA target site with high AGO accessibility in SARS-CoV-2 as determined using P_exp_ with the optimised parameter (*u* = 8, *L* = 20). (**c,d**) The positional conservation rate of SARS-CoV-2 gRNAs from multiple alignments of the reported sequencing results (n = 5475); highlighted by red dots for the nsp12 region (**c**). Delineated potent siRNA target sites with seed matches of antifibrotic miRNAs are indicated (**d**) by the same colour in the legend (upper right panel); red arrow, a siRNA target in nsp12 (RdRP) with miR-27a seed (27/RdRP); yellow shade for the nsp12 region. (**e**) List of 13 designed siRNA sequences that potently target the putative AGO accessible regions (P_exp_ > 0.1), containing seed (more than 6mers within positions 2–8) sequences of antifibrotic miRNAs, and overlapping with invariant regions in the SARS-CoV-2 sequences (n = 5475); locations of the target sites and seed sequences (SARS-CoV-2; NC_045512); exposure probability (P_exp_) of the seed sites (*u* = 8, *L* = 20); yellow shade, 27/RdRP. (**f-i**) Experimental validation of miRNA-mimicking siRNAs designed in (**e**); 3 siRNAs highlighted with a shade. For the miR-151 seed containing siRNA that targets nsp3 (151/nsp3), luciferase reporter assays were conducted for a single siRNA target site with ± 45 bp flanking sequences (**f**, upper panel) and seed-mediated miRNA-like repression with five 6mer seed sites (**f**, lower panel); the activity was normalised by firefly luciferase with no site (relative activity), estimating IC_50_ in HeLa cells. The same luciferase assays were performed for on-target activity of the miR-193a-5p seed containing siRNAs that target nsp3 (193/nsp3; g) and nsp5 (193/nsp5; **h**). But for miRNA-like activity, a miR-193a-5p target transcript, COL1A1 (**i**, left panel) was used to measure the abundance depending on 193/nsp3, 193/nsp5, and miR-193a-5p expression in MRC-5 cells by performing qPCR (**i**, right panel); normalised by GAPDH mRNA and represented relatively to NT (non-targeting miRNA; negative control) transfection (relative mRNA level). *P*-values from *t*-test, two-sided; *P < 0.05; relative to NT; n ≥ 3; repeated with biologically independent samples; graphs, mean; error bars, SD.

Based on the defined AGO binding in nsp12 (Fig. 2a), we further predicted the AGO accessibility of every position in the SARS-CoV-2 gRNA by calculating P_exp_ with the optimised parameter (*u* = 8, *L* = 20; Fig. 2b). RNA viruses are frequently mutated during viral replication and propagation; however, the regions that play critical roles are relatively conserved^4^. Thus, siRNA is required to target invariant regions of viral gRNA to guarantee its consistent effectiveness in the human spread. Accordingly, sequence conservation rates were considered through an analysis of multiple sequence alignments of the SARS-CoV-2 sequences reported in human infections (n = 5475; Fig. 2c). Ultimately, this revealed several mutation hotspots that should be avoided in siRNA targeting.

Because miRNAs also mediate AGO-dependent target suppression, siRNAs inevitably exert miRNA-like off-target repression through seed matches. To advantageously arrange such miRNA-like activity to prevent fatal pulmonary fibrosis in COVID-19, we designed siRNAs with seed sequences of the antifibrotic miRNAs (miR-27a, miR-193-5p, miR-486, miR-151, and miR-455), which were previously identified by their downregulation in lung fibrosis models^17^. After selecting the AGO-accessible regions (P_exp_ > 0.1; Fig. 2b) that have no reported sequence variation in SARS-CoV-2 (100% conservation in seed sites (positions 2–8); Fig. 2c), 13 siRNA sequences were determined (Fig. 2d,e) to have seed sequences of miR-27 (n = 2), miR-193-5p (n = 3), miR-486 (n = 2), miR-151 (n = 4), and miR-455 (n = 2). These were expected to function as siRNAs that exhibit antifibrotic miRNA activity and suppress SARS-CoV-2.

### Validation of siRNAs targeting SARS-CoV-2 with antifibrotic miRNA activity

Among the 13 designed siRNAs (Fig. 2e), we initially validated 3 siRNAs with high AGO accessibility (P_exp_ > 0.5) for their target repression and miRNA-like activity; a miR-151 mimicking siRNA that targets nsp3 (named “151/nsp3”, P_exp_ = 0.55; Fig. 2f), and miR-193a-5p mimicking siRNAs that target nsp3 (193/nsp3, P_exp_ = 0.65; Fig. 2g,i) and nsp5 (193/nsp5, P_exp_ = 0.64; Fig. 2h,i). Using luciferase reporter assays, we measured the half maximal inhibitory concentration (IC_50_) that suppressed target sites containing flanking sequences (± 45 nucleotides) and confirmed that all of these 3 siRNAs can efficiently silence transcripts with SARS-CoV-2 target sites (IC_50_[151/nsp3] = 0.31 nM, Fig. 2f, upper panel; IC_50_[193/nsp3] = 0.34 nM, Fig. 2g; IC_50_[193/nsp5] = 0.38 nM; Fig. 2h). For 151/nsp3, a luciferase reporter with miR-151 seed sites was used to verify its miRNA-like activity (IC_50_ = 5.59 nM, Fig. 2f, lower panel). To examine a fibrosis-related target gene for antifibrotic miRNA-like activity, we selected COL1A1, the major component of type I collagen^35^, of which the 3′UTR contains seed sites of miR-193a-5p (positions 1–8; Fig. 2i, left panel). Therefore, we investigated the miRNA-like activity of 193/nsp3 and 193/nsp5 by measuring the COL1A1 transcript level as a validated target of miR-193a-5p^36^, confirming that they can repress COL1A1 mRNA to a comparable extent that was silenced by transfecting miR-193a-5p into the human fibroblast lung cells, MRC-5 (Fig. 2i, right panel). Altogether, we validated effective target repression mediated by several designed siRNA sequences that target SARS-CoV-2 as well as mimic antifibrotic miRNAs.

**Figure 3:**
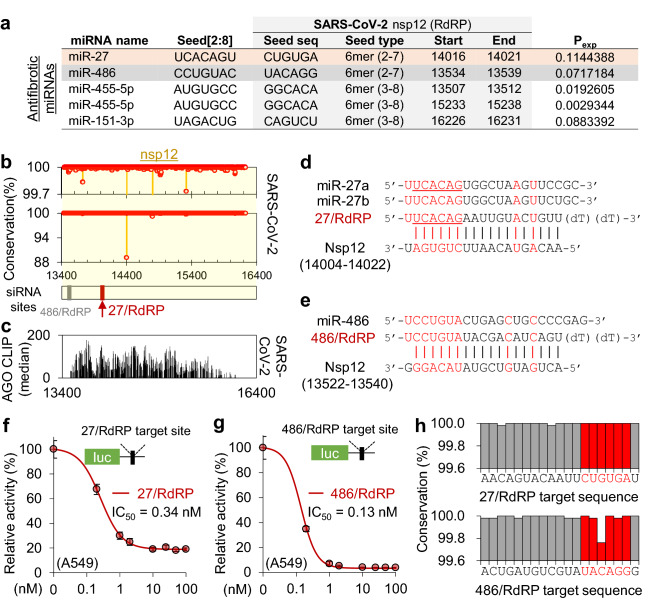
AGO-accessible siRNAs targeting the nsp12 of SARS-CoV-2 with antifibrotic miRNA seeds. (**a**) Seed sites of previously reported antifibrotic miRNAs located in nsp12 regions of SARS-CoV-2. Considering the AGO accessibility threshold (P_exp_ > 0.1), only the miR-27 seed site was selected in Fig. 2, but all seed sites of the antifibrotic miRNAs are presented in the table. (**b**) The positional conservation rate of SARS-CoV-2 gRNAs in the nsp12 region among the cases of human infection (n = 5475); highlighted by red dots and its zoom-in display (upper panel); red arrow, the target position of 27/RdRP; grey arrow, the target position of 486/RdRP. (**c**) The inferred AGO accessibility derived from AGO CLIP data of RNA viruses. (**d,e**) Sequences of 27/RdRP with miR-27a and miR-27b (**d**), and 486/RdRP with miR-486 (**e**), aligned with its binding site in the SARS-CoV-2 nsp12. (**f,g**) Luciferase reporter assays for a single perfect target site for 27/RdRP (**f**) or 486/RdRP (**g**) in the renilla luciferase 3′UTR, of which the activity was normalised by firefly luciferase with no site (relative activity) and used to calculate the IC_50_ in human epithelial lung cell A549; the same experiment was conducted with different cell lines (Supplementary Fig. S3a). (**h**) Positional sequence conservation of the 27/RdRP (upper panel) and 486/RdRP sites (lower panel), calculated from multiple sequence alignment of 5475 SARS-CoV-2 sequences in the GenBank. Of note, there is no variation (100% conservation) in every position in the seed (highlighted in red) of 27/RdRP; however, there is a 0.3% variation at position 5 of the seed site in 486/RdRP.

Because the RdRP protein, encoded by nsp12, is essential for viral replication, it has often been a therapeutic target of antiviral drugs. Therefore, we focused on a siRNA that targets nsp12 with a miR-27 seed site (named “27/RdRP”; Fig. 2e), which was notably located on the peak of the AGO-bound region and had no sequence variation (Fig. 2c–e). Although only 27/RdRP was chosen to be AGO accessible in the nsp12 region (P_exp_ > 0.1; Fig. 2b), four additional siRNAs could be designed to have target sites that match the seed of the antifibrotic miRNAs (miR-27a, miR-486, miR-151, and miR-455; Fig. 3a) regardless of AGO accessibility (Fig. 3b,c). In addition to 27/RdRP (Fig. 3d), the siRNA with the miR-486 seed (486/RdRP; Fig. 3e) showed marginal AGO accessibility immediately below the threshold (P_exp_ = 0.072; Fig. 3a), which could be worthy of experimental testing for silencing activity. In the luciferase reporter assays with corresponding siRNA target sites, high efficiency of target repression was observed for both 27/RdRP (IC_50_ = 0.34 nM in A549 cells, Fig. 3f; IC_50_ = 0.28 nM in HeLa cells, Supplementary Fig. S3a) and 486/RdRP (IC_50_ = 0.13 nM in A549 cells, Fig. 3g). However, the target site of 486/RdRP was found to contain critical variations in SARS-CoV-2 sequences within its seed matched region (Fig. 3h), which was reported to be detrimental to RNAi activity.

### Transcriptome-wide validation of 27/RdRP for antifibrotic miRNA-like activity

**Figure 4:**
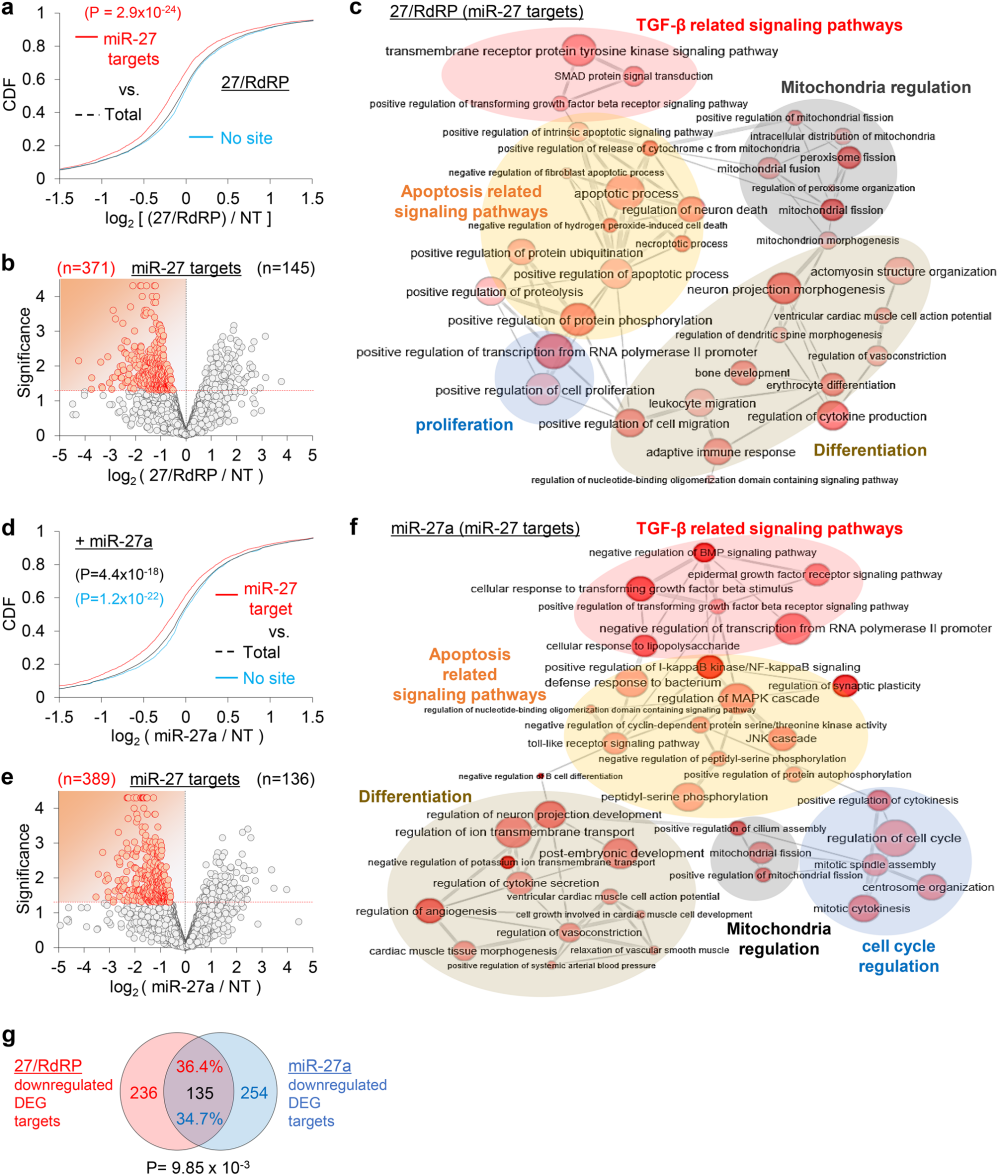
Transcriptome-wide assessment of miRNA-like activity in 27/RdRP. (**a**) RNA-Seq analyses were performed in 27/RdRP-transfected A549 cells. CDF analysis using RNA-Seq for putative miR-27 targets, that harbour 7mer-A1 seed sites (an exact match to positions 2–7 followed by an ‘A’ at position 1) in their 3′UTRs, according to their fold changes (log2 ratio; relative to the non-targeting miRNA, ‘NT’, negative control); RPKM values from Cufflinks; *P*-values from Kolmogorov–Smirnov test (two-sided) relative to the total transcripts (Total); ‘No site’, transcripts with no 6mer seed matches (positions 2–8). (**b**) Volcano plot analyses of the putative miR-27 targets, which contain 6mer seed site (positions 2–7) in 3′UTR, in the presence of 27/RdRP expression; significance, − log_10_ (*P*-value); downregulated differentially expressed genes (DEG, *P* < 0.05; Cuffdiff), highlighted in red. (**c**) GO analysis results of the downregulated DEGs in 27/RdRP transfected A549 cells, which are presented as networks of enriched biological process terms (EASE score < 0.2, DAVID; node size and colour intensity inversely correlate with *P*-value). Only clusters of graphs with high connections are displayed. (**d–f**) Same analyses as conducted in (**a**–**c**) except for miR-27a-transfected A549 cells. (**g**) Significant overlap (upper panel, n = 489) in the differentially downregulated targets between 27/RdRP (**b**) and miR-27a (**e**) expression (*P* = 9.85 × 10^–3^; chi-square test, relative to random shuffling in Supplementary Fig. S3c); details in the Material and methods section.

After confirming the siRNA activity of 27/RdRP, we verified its transcriptome-wide miRNA-like activity by performing RNA sequencing (RNA-Seq; Supplementary Fig. S3b). In the CDF analyses (Fig. 4a), the putative miR-27 target transcripts, which harbor the seed sites in the 3′ untranslated region (3′UTR), were significantly downregulated when 27/RdRP was transfected into human epithelial lung cell, A549 (*P* = 2.9 × 10^–24^, Kolmogorov–Smirnov test, relative to control transfection (non-targeting miRNA; “NT”)). Among the putative miR-27 targets (miR-27 seed sites in 3′UTRs) in differentially expressed genes (DEGs; *P* < 0.05, Cuffdiff), most were downregulated (log_2_ fold change < 0) by 27/RdRP (n = 371 vs. 145; Fig. 4b). Gene ontology (GO) analysis revealed that the miR-27 targets (downregulated DEGs with miR-27 seed sites in 3′UTRs) of 27/RdRP were found to function in lung fibrosis, mediated by TGF-β signalling pathways, in conjunction with apoptosis, differentiation, mitochondrial regulation, and proliferation (Fig. 4c). Downregulation of miR-27 targets was also induced by miR-27a expression in A549 cells (Fig. 4d,e) and was found to be enriched in similar functional networks regulated by 27/RdRP (Fig. 4f).

The differentially downregulated targets (log_2_ fold change < 0, miR-27 seed sites in 3′UTRs, and *P* < 0.05) between 27/RdRP and miR-27a (Fig. 4b,e) was significantly overlapped (n = 135; *P* = 9.85 × 10^–3^, chi-square test; Fig. 4g) relative to random control (1000 shuffling; Supplementary Fig. S3c) and enriched in the gene ontology function related to fibrosis (e.g. TGF-β signalling pathways, apoptosis, differentiation, mitochondria regulation, and cell cycle regulation; Supplementary Fig. S3d). The same significant overlap (*P* = 3.23 × 10^–81^) was also observed in downregulated DEG targets that only selected by fold change (log_2_ fold change < -0.5 and miR-27 seed sites in 3′UTRs; Supplementary Fig. S3e and Supplementary Table S2). Of note, although miR-27 targets were selected simply by checking seed sites in 3′UTRs, which possibly contained false positive, significant propensity of target repression was observed in 27/RdRP transfection (Fig. 4a,b) as well as in miR-27a transfection (Fig. 4d,e). Additionally, predominant downregulation of putative miR-27 targets (seed sites in 3′UTRs) in DEGs depending on 27/RdRP or miR-27a expression (Fig. 4b,e) reflects primary effects as miR-27 targets, in contrast to upregulated DEGs which may reflect the secondary effect from target repression. The consensus secondary effect, that overlaps between 27/RdRP and miR-27a, could be examined in part as only looking at overlapping downregulation and their gene ontologies. These results demonstrated that siRNAs targeting SARS-CoV-2 could direct the miRNA-like off-target effects to the way that could potentially suppress pro-fibrotic pathways by containing the same seed sequences of antifibrotic miRNAs. Overall, miRNA-like activity of 27/RdRP could be demonstrated to globally function like miR-27a, potentially conferring antifibrotic activity in lung cells, where one such regulation could be mediated by attenuating the TGF-β response.

### Validation of the antifibrotic siRNA activity of 27/RdRP targeting SARS-CoV-2

**Figure 5:**
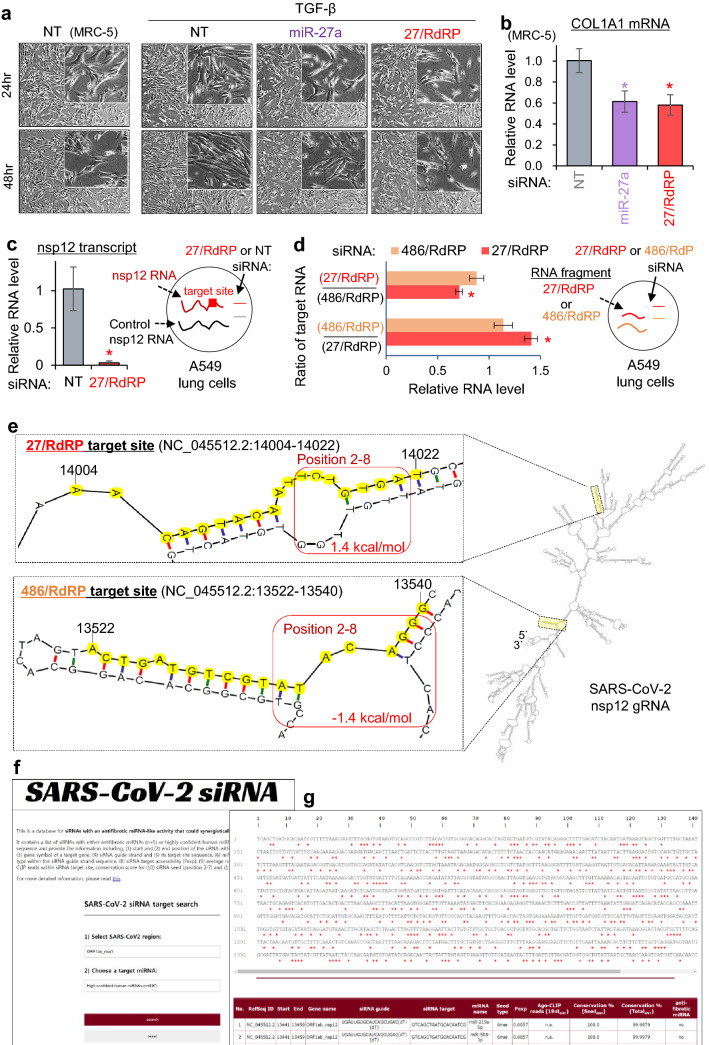
Validation of 27/RdRP repressing nsp12 of SARS-CoV-2 with antifibrotic miRNA activity. (**a**) Morphology of human lung fibroblast cell line MRC-5 after treatment with 2.5 ng/mL TGF-β. Fibrotic differentiation of MRC-5 was examined after transfection of non-targeting control (NT), miR-27a, and 27/RdRP; 100 times magnified bright-field microscope images are presented; 400 times, inner set. Of note, fibrotic differentiation with long and thin morphology was induced by TGF-β treatment, but substantially attenuated by expressing 27/RdRP or miR-27a. (**b**) The relative level of COL1A1 mRNA after 27/RdRP or miR-27a was introduced into MRC-5 cells; the level was normalised to that of GAPDH mRNA and presented as relatively to NT (non-targeting miRNA; negative control) transfection. Of note, owing to the extremely low expression of COL1A1 mRNA in A549 cells (Supplementary Fig. S3d), qPCR experiments were only conducted in the MRC-5 cells. Although the change in morphology in (**a**) was marginal, repression of a collagen gene, COL1A1, was significant (**b**). (**c**) The abundance of the SARS-CoV-2 nsp12 transcript, which was transfected into A549 cells together with the control nsp12 transcript (codon-optimised nsp12 transcript; “ + Control”), was measured in the presence of 27/RdRP expression. The relative RNA level of the nsp12 transcript was measured by the abundance of the co-transfected control transcript. Of note, both the nsp12 and the control transcripts, generated from in vitro transcription, have the same length and capability to be translated into the nsp12 protein. However, the control transcript contains no target site of 27/RdRP due to the difference in RNA sequences. (**d**) Same experiments as performed in (**c**) except that the nsp12 RNA fragments were co-transfected with 27/RdRP or 486/RdRP target site to relatively measure the ratio of the target RNA (relative RNA level) in the presence of 27/RdRP or 486/RdRP. All *P*-values from the *t*-test, two-sided; *P < 0.05; relative to NT; n ≥ 3; repeated with biologically independent samples; graphs, mean; error bars, SD. (**e**) Predicted RNA secondary structure of the SARS-CoV-2 nsp12 (right panel; predicted via the mfold program^32^) with the zoomed view of 27/RdRP (upper left panel) and 486/RdRP (lower left panel) target sites; target site sequences are highlighted in yellow. The minimum free energy of 27/RdRP and 486/RdRP seed region (positions 2–8) in the secondary structure was calculated using the RNAduplex program^33^ (highlighted with a red box). (**f,g**) The web server for SARS-CoV-2 siRNAs with searchable functionality (**f**) for specified miRNAs including antifibrotic miRNAs (n = 5) and other high confident miRNAs (n = 897), providing exposure probability (P_exp_), and inferred AGO binding in output results (**g**). Detail information is available online (http://clip.korea.ac.kr/covid19).

It is well known that fibrosis is critically regulated by the TGF-β signalling pathway, featuring collagen accumulation in the extracellular matrix^35^. Since we found that 27/RdRP could globally suppress miR-27 target mRNAs that mainly function in TGF-β signalling (Fig. 4c), we examined the effect of TGF-β treatment on fibroblasts in the presence of 27/RdRP expression (Fig. 5a). As previously reported, TGF-β treatment induced differentiation of lung fibroblast cells, MRC-5, but the differentiation was attenuated by transfecting 27/RdRP as well as by transfecting miR-27a (Fig. 5a). Furthermore, by expressing 27/RdRP, COL1A1 transcript, which encodes the major component of type I collagen and harbours a miR-27a seed site in the 3′UTR (positions 3–8; Fig. 2i, left panel), was significantly reduced as observed in miR-27a transfection (Fig. 5b), demonstrating that 27/RdRP could prevent pulmonary fibrosis by functioning as an antifibrotic miR-27a.

Finally, to validate 27/RdRP for its silencing activity against SARS-CoV-2, the same nsp12 transcript of SARS-CoV-2 was synthesised in vitro and ectopically introduced into A549 lung cells as a mimic of viral infection (Fig. 5c,d). Although high efficiency of target repression was observed for 27/RdRP and 486/RdRP (Fig. 3f,g), it could be simply because the luciferase reporters contained only 20nt-long target sites without any flanking sequences, which may differentially affect local RNA folding. Thus, we validated those sites in the context of SARS-CoV2 sequences again. By quantifying the transfected nsp12 transcript relative to the control transcript (codon-optimised nsp12 construct with no 27/RdRP site), 27/RdRP was validated to specifically and remarkably reduce the nsp12 transcript (Fig. 5c). In addition, 27/RdRP showed significant repression of the RNA fragment containing the 27/RdRP target site with only ± 43 nucleotides flanking regions, even though the activity was relatively measured by the abundance of cognate RNA fragments suppressed by 486/RdRP (Fig. 5d). Although both 27/RdRP and 486/RdRP showed comparable repressive activity in the luciferase reporters that contained only the target site (20 nucleotides) without flanking sequences (Fig. 3f,g), 486/RdRP showed negligible silencing activity relative to 27/RdRP in the context of neighbouring sequences, which can affect local RNA folding (entire transcript or with ± 43 nucleotides from the target sites; Fig. 5c,d). This discrepancy in 486/RdRP could be explained by the lack of AGO association inferred from CLIP data (Fig. 2) and lower exposure probability (P_exp_ < 0.1; Fig. 3a) of local RNA folding. Indeed, in the predicted RNA secondary structure of SARS-CoV-2 nsp12 (predicted by mfold program^32^; Fig. 5e), 27/RdRP seed site in the local base pairing is very unstable (1.4 kcal/mol, estimated by RNAduplex program^33^) in contrast to stable secondary structure in 486/RdRP seed site (-1.4 kcal/mol). Overall, we validated the robust activity of 27/RdRP in repressing SARS-CoV-2 and preventing lung fibrosis via its antifibrotic miR-27 activity. In addition, to facilitate the use of all putative siRNAs designed here for targeting SARS-CoV-2, we established a webserver (http://clip.korea.ac.kr/covid19) that provides sequence information of siRNAs which contain seed sequences of not only antifibrotic miRNAs (n = 5) but also high-confident human miRNAs (n = 897) with search functionality (Fig. 5f). Total 14,028 target sites (antifibrotic miRNAs, n = 75; high-confident human miRNAs, n = 13,953) were identified in SARS-CoV-2 gRNA, offering exposure probability (P_exp_), and inferred AGO binding regions (Fig. 5g).

## Conclusion

To design potent siRNA sequences against SARS-CoV-2, we attempted to infer putative AGO-accessible regions in SARS-CoV-2 gRNA by initially reanalysing AGO CLIP data from other RNA viruses in the conserved nsp12 region encoding RdRP. To expand the analysis from the conserved region, we applied the local RNA folding method by optimising the parameters with the inferred AGO binding in nsp12, calculated exposure probability (P_exp_), and designed potent siRNAs that targeted the predicted AGO-accessible regions in SARS-CoV-2. To treat COVID-19 by silencing SARS-CoV-2 as well as by inhibiting the progression to fatal lung fibrosis, we utilised miRNA-like off-target activity of siRNAs by adopting seed sequences from antifibrotic miRNAs. Avoiding sequence variants in SARS-CoV-2, we designed 13 antifibrotic miRNA-mimicking siRNAs that target SARS-CoV-2 and validated some of their silencing activity as dual players. Among them, 27/RdRP was functionally validated to target the nsp12 region of SARS-CoV-2, confirmed for its transcriptome-wide activity, similar to antifibrotic miR-27a, and experimentally proven to suppress TGF-β-induced lung fibrosis and a collagen-producing gene, COL1A1, in human lung cells. Our current work provides bioinformatics approaches to design robust and synergistic siRNA drugs that potentially inhibit SARS-CoV-2 and attenuate fatal pulmonary fibrosis in COVID-19, offered as a web resource (http://clip.korea.ac.kr/covid19). Although further experiments are needed for clinical application, we hope that the siRNA sequences delineated here will offer more options for the rapid development of siRNA drugs to treat and save patients with COVID-19.

## Supplementary Information

Supplementary Information.

## Data Availability

The datasets analysed during the current study are available in the GEO database (https://www.ncbi.nlm.nih.gov/geo/; GSE76967) and SRA repository (https ://www.ncbi.nlm.nih.gov/sra; SRP270828).
