# Supplementary material for: AGO CLIP-based imputation of potent siRNA sequences targeting SARS-CoV-2 with antifibrotic miRNA-like activity
Source: Sci Rep. 2021 Sep 27;11:19161. doi: 10.1038/s41598-021-98708-z (PMC8476540; doi:10.1038/s41598-021-98708-z)
Supplement: Supplementary file 1 — Supplementary Information. [file 41598_2021_98708_MOESM1_ESM.pdf]

Supplementary information

AGO CLIP-based imputation of potent siRNA sequences targeting SARS-CoV-2 with antifibrotic miRNA-like activity

Seung Hyun Ahn[1,3], Dowoon Gu[1,3], Yongjun Koh[2], Hye-Sook Lee[1] & Sung Wook Chi[1,*]

[1]Department of Life Sciences, Korea University, Seoul, Korea. [2]Division of Biotechnology, College of Life Sciences and Biotechnology, Korea University, Seoul, Korea. [3]These authors contributed equally: Seung Hyun Ahn and Dowoon Gu. *email: chi13@korea.ac.kr

Supplementary Table S1

Name	Description	Sequences
NT	Guide	5’p UCACAØCCUCCUAGAAAGA (dT)(dT) 3’
(MI0000038)	Passenger	5’p UCUUUØUAGGAGGUUGUGA (dT)(dT) 3’
miR-27a	Guide	5’p UUCACAGUGGCUAAGUUCC(dT)(dT) 3’
(MI0000085)	Passenger	5’p mGmGAACUUAGCCACUGUGAA(dT)(dT) 3’
miR-193a-5p	Guide	5’p UGGGUCUUUGCGGGCGAGA (dT)(dT) 3’
(MIMAT0004614)	Passenger	5’p UCUCGCCCGCAAAGACCCA(dT)(dT) 3’
151/nsp3	Guide	5'p UAAGACUGGUCAGUAGGAU(dT)(dT) 3'
	Passenger	5'FAM AUCCUACUGACCAGUCUUC(dT)(dT)3'
193/nsp3	Guide	5'p UUGGUCUUUUAAACUGUAG(dT)(dT) 3'
	Passenger	5'FAM CUACAGUUUAAAAGACCAA(dT)(dT) 3'
193/nsp5	Guide	5'p AUGGUCUUGUGUUAGAGGU(dT)(dT) 3'
	Passenger	5'FAM ACCUCUAACACAAGACCAU(dT)(dT) 3'
27/RdRP	Guide	5’p UUCACAGAAUUGUACUGUU(dT)(dT) 3’
	Passenger	5’p mAmACAGUACAAUUCUGUGAA(dT)(dT) 3’
486/RdRP	Guide	5’p UCCUGUAUACGACAUCAGU(dT)(dT) 3’
	Passenger	5’p mAmCUGAUGUCGUAUACAGGA(dT)(dT) 3’

Supplementary Table S1. siRNAs and miRNAs used in this study. “Ø” indicates abasic deoxynucleotide (dSpacer), “m” denotes 2’-O methyl modification, and “(dT)” represents thymidine deoxynucleotide. “FAM” indicates 6-fluorescein amidite. All RNAs were synthesized with 5’ phosphate (5’p). Of note, non-targeting miRNA (NT) was derived from cel-miR-67 sequence (C. elegans-specific miRNA) and synthesized as a form of siRNA, which harbor two thymidine deoxynucleotides (dT) overhangs and contain abasic pivot (abasic deoxynucleotide, dSpacer (Ø), at position 6) in both strands to avoid miRNA-like off-target repression, as previously reported[1]. Moreover, the passenger strands of siRNAs contain 2’-O methylation at positions 1 and 2 (2’OMe) or FAM at 5’ end in the form of siRNA, preventing seed-mediated repression from the passenger strand[2].

Supplementary Table S2

Enrichment Score: 0.6882429821410878

Term	description	%	P-value	Fold Enrichment
GO:1990001	inhibition of cysteine-type endopeptidase activity involved in apoptotic process	0.7009	0.0350	9.6186
GO:0090307	mitotic spindle assembly	0.7009	0.4482	1.9900
GO:0043066	negative regulation of apoptotic process	3.2710	0.5492	1.0687

Enrichment Score: 0.22745527382629274

Term	description	%	P-value	Fold Enrichment
GO:0042787	protein ubiquitination involved in ubiquitin-dependent protein catabolic process	1.8692	0.4390	1.2719
GO:0000209	protein polyubiquitination	1.8692	0.5946	1.0993
GO:0043161	proteasome-mediated ubiquitin-dependent protein catabolic process	1.8692	0.7960	0.8947

Enrichment Score: 0.2026436186035105

Term	description	%	P-value	Fold Enrichment
GO:0000086	G2/M transition of mitotic cell cycle	1.8692	0.3274	1.4250
GO:0007067	mitotic nuclear division	2.3364	0.7687	0.9161
GO:0051301	cell division	2.1028	0.9800	0.6206

Enrichment Score: 0.19995661378199037

Term	description	%	P-value	Fold Enrichment
GO:0000122	negative regulation of transcription from RNA polymerase II promoter	4.9065	0.4837	1.0773
GO:0006351	transcription, DNA-templated	10.5140	0.6685	0.9694
GO:0045892	negative regulation of transcription, DNA-templated	3.0374	0.7771	0.9061

Enrichment Score: 0.12605122814189287

Term	description	%	P-value	Fold Enrichment
GO:0006406	mRNA export from nucleus	1.1682	0.6852	1.0807
GO:0031124	mRNA 3'-end processing	0.7009	0.6856	1.2825
GO:0006369	termination of RNA polymerase II transcription	0.7009	0.8109	0.9950
GO:0000398	mRNA splicing, via spliceosome	2.1028	0.8221	0.8657

Enrichment Score: 0.10135290136731931

Term	description	%	P-value	Fold Enrichment
GO:0006614	SRP-dependent cotranslational protein targeting to membrane	1.4019	0.5131	1.2684
GO:0006364	rRNA processing	2.3364	0.7116	0.9667
GO:0019083	viral transcription	1.1682	0.8188	0.8906
GO:0000184	nuclear-transcribed mRNA catabolic process, nonsense-mediated decay	1.1682	0.8450	0.8512
GO:0006412	translation	1.4019	0.9850	0.5576
GO:0006413	translational initiation	0.7009	0.9908	0.4580

Supplementary Table S2. Gene ontology analysis results for overlapping miR-27 target genes, downregulated both by 27/RdRP and miR-27a expression. Clustered GO analysis results for overlapping downregulated genes in both 27/RdRP and miR-27a expression (n=489, Supplementary Fig. 3c), measured in A549 cells by RNA-Seq. Enrichment scores were calculated relatively to total expressed transcripts (RPKM>0 and log2(volume)>2) using DAVID program (https://david.ncifcrf.gov/) and only the clusters with high enrichment score were represented (enrichment score > 0.1). Of note, gene ontologies related with fibrosis were enriched (e.g. apoptosis, mitotic spindle, protein ubiquitination, cell cycle and RNA regulation).

Supplementary Figure S1

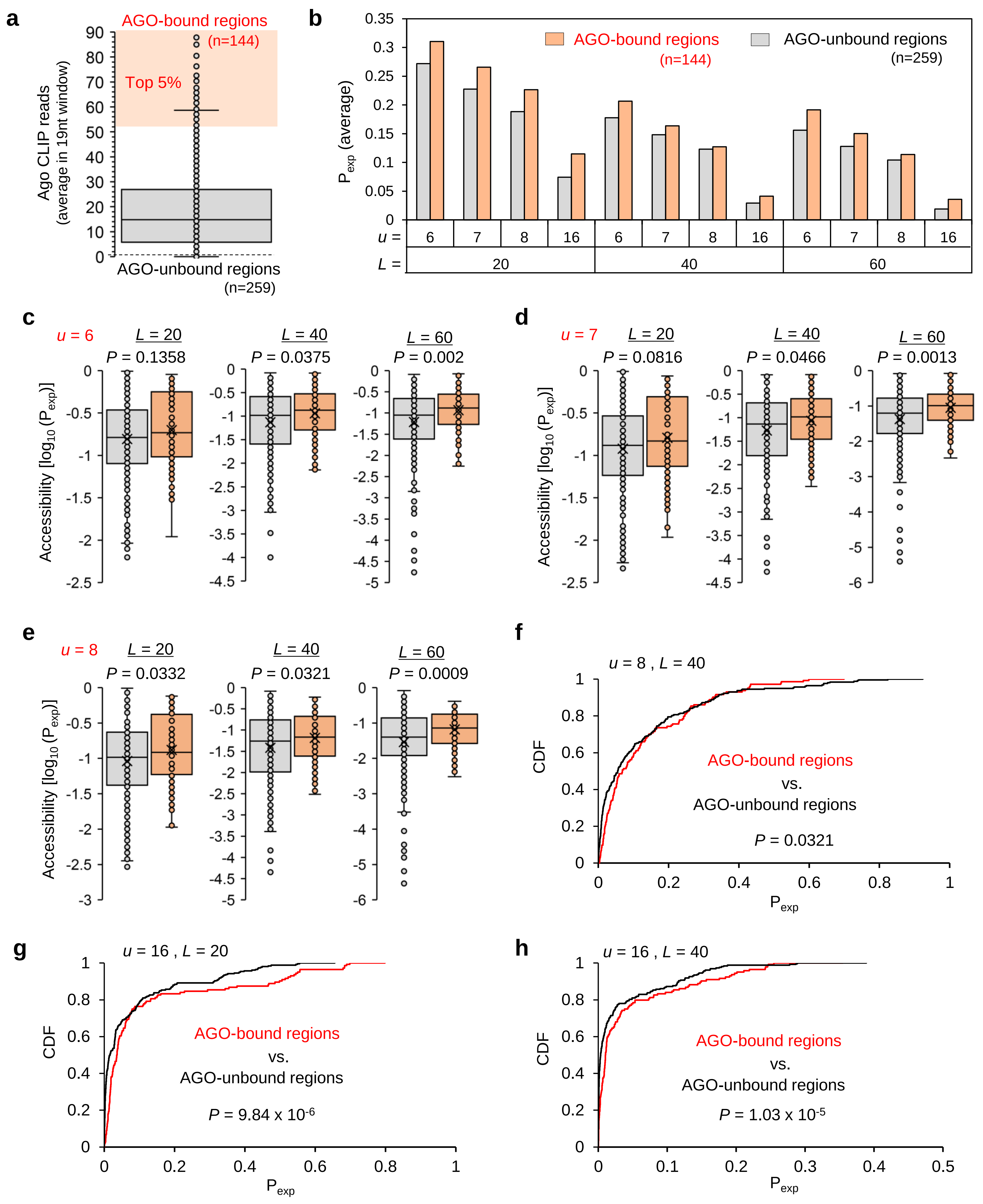

Supplementary Figure S1. Optimization of the accessibility parameters in RNA local folding prediction based on the inferred AGO-bound regions in SARS-CoV-2 nsp12. (a) Ranking of every site in SARS-CoV-2 nsp12 region, derived from meta-analyses of AGO CLIP data from related RNA viruses. A set of high confident AGO-bound regions was defined by selecting the top 5% of AGO CLIP reads (average read-count in the 19 nucleotides window; n=144). AGO-unbound regions were selected as the sites with neither AGO CLIP reads nor reproducible reads across different RNA viruses (biological complexity = 1; n=259). **(b)** Partition function for all local structures within the window (80 nucleotides, W=80) was calculated to predict exposure probability (P_{exp}) using RNAplfold[3] under different constraints; length of a region for accessibility (*u* = 6, 7, 8, and 16); range of nucleotides allowing local base pairing (*L* = 20, 40, and 60). The average P_{exp} was compared between AGO-bound and AGO-unbound regions as defined in (a). **(c-e)** Accessibility of the target sites ($\log_{10}(P_{exp})$) was calculated under different ranges of local base-pairing (*L* = 20, 40, and 60) for the length of the seed sites (*u* = 6, 7 and 8) including the 6mer (c), 7mer (d) and 8mer site (e) in position 2-9; AGO-bound vs. AGO-unbound regions. **(f-h)** Cumulative distribution function (CDF) analyses of exposure probability (P_{exp}) depending on different local RNA folding parameters; CDF results of P_{exp} in the 8mer seed sites (*u* = 8) were compared to the different ranges of local base pairing (*L* = 20 vs. 40; Fig. 1e vs. (f)). CDF results of P_{exp} in 16 nucleotides from the 5' end of siRNA (*u* = 16) were examined between *L* = 20 and *L* = 40 ((g) vs. (h)) as previously reported[3]; P-values (*P*), Wilcoxon rank sum test (two-sided).

Supplementary Figure S2

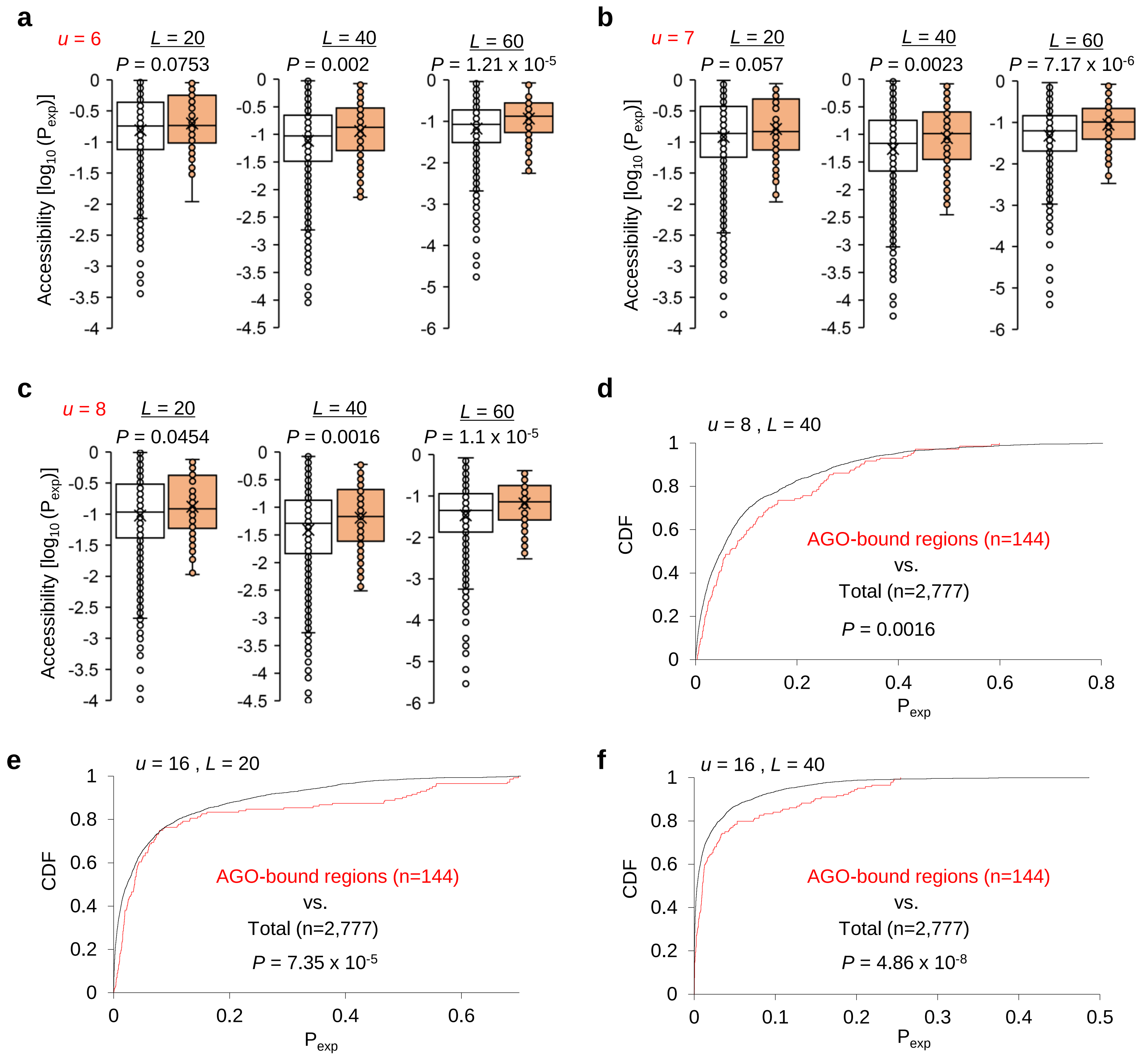

Supplementary Figure S2. Optimization of accessibility parameters in local RNA folding using AGO-bound regions relative to total regions in SARS-CoV-2 nsp12. (a-c) Accessibility of the target sites ($\log_{10}(P_{exp})$) was calculated as conducted in Supplementary Fig. 1c-e except using total regions as control. **(d-f)** Cumulative distribution function (CDF) analyses of exposure probability (P_{exp}) depending on different local RNA folding parameters as performed in Supplementary Fig. 1f-h except using total regions as control.

Supplementary Figure S3

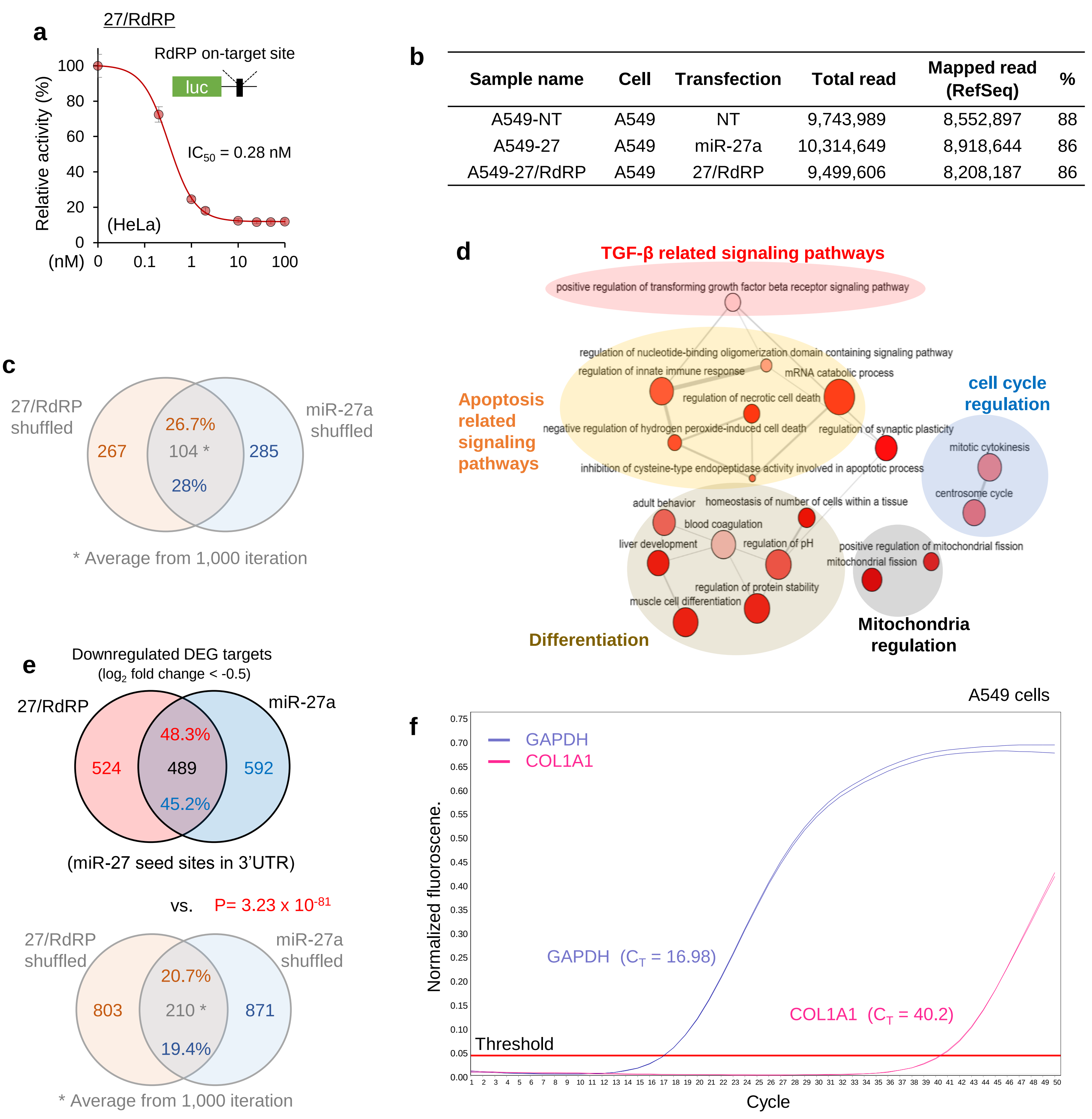

Sample name	Cell	Transfection	Total read	Mapped read (RefSeq)	%
A549-NT	A549	NT	9,743,989	8,552,897	88
A549-27	A549	miR-27a	10,314,649	8,918,644	86
A549-27/RdRP	A549	27/RdRP	9,499,606	8,208,187	86

Supplementary Figure S3. Validation of 27/RdRP siRNA targeting SARS-CoV-2 nsp12 with miR-27-like activity (a) Luciferase reporter assays with on-target site as employed and represented in Figure 3f, except performing in HeLa cells. The target site (20 nucleotides) with no flanking sequences is located in 3'UTR of renila luciferase, of which activity was normalized by firefly luciferase with no site (relative activity) and used to calculate the IC_{50}. **(b)** Mapping rates of the RNA-Seq reads obtained after transfection of NT, miR-27a and 27/RdRP into A549 cells; results from running the STAR program with RefSeq annotation. **(c)** The random shuffling control (n=1,000) used in overlap analysis of 27/RdRP and miR-27a downregulated targets (Fig. 4g). **(d)** GO analysis results of the overlapping downregulated DEGs between 27/RdRP and miR-27a (Fig. 4g), which are presented as networks of enriched biological process terms (DAVID; node size and colour intensity inversely correlate with P-value). Only clusters of graphs with high connections are displayed. **(e)** Significant overlap (upper panel, n=489) in the differentially downregulated targets (log_2 fold change < -0.5 and miR-27 seed sites (6mers in position 2-8) in 3'UTRs) between 27/RdRP and miR-27a expression ($P=3.23 \times 10^{-81}$; Chi-squared test, relative to random shuffling; lower panel, n=1,000). **(f)** Amplification plots from qPCR for GAPDH and COL1A1 mRNAs, conducted in A549 cells. Cycle threshold (C_T) for GAPDH mRNA is 16.98 and for COL1A1 mRNA is 40.20. Of note, based on C_T value, COL1A1 gene seems to be not expressed in A549 cells.

References

1. Lee, HS, Seok, H, Lee, DH, Ham, J, Lee, W, Youm, EM, et al. (2015). Abasic pivot substitution harnesses target specificity of RNA interference. Nat Commun 6: 10154.

2. Jackson, AL, Burchard, J, Leake, D, Reynolds, A, Schelter, J, Guo, J, et al. (2006). Position-specific chemical modification of siRNAs reduces "off-target" transcript silencing. RNA 12: 1197-1205.

3. Tafer, H, Ameres, SL, Obernosterer, G, Gebeshuber, CA, Schroeder, R, Martinez, J, et al. (2008). The impact of target site accessibility on the design of effective siRNAs. Nat Biotechnol 26: 578-583.
